# Identify CRNDE and LINC00152 as the key lncRNAs in age-related degeneration of articular cartilage through comprehensive and integrative analysis

**DOI:** 10.7717/peerj.7024

**Published:** 2019-05-28

**Authors:** Pengfei Hu, Fangfang Sun, Jisheng Ran, Lidong Wu

**Affiliations:** 1 Department of Orthopedic Surgery, the Second Affiliated Hospital, School of Medicine, Zhejiang University, Hangzhou, Zhejiang, China; 2 Orthopedics Research Institute of Zhejiang University, Hangzhou, Zhejiang, China; 3 Key Laboratory of Cancer Prevention and Intervention, China National Ministry of Education, the Second Affiliated Hospital, Cancer Institute, School of Medicine, Zhejiang University, Hangzhou, Zhejiang, China

**Keywords:** Osteoarthritis, CRNDE, LINC00152, KEGG pathway, DEGs, GO annotation

## Abstract

**Background:**

Osteoarthritis (OA) is one of the most important age-related degenerative diseases, and the leading cause of disability and chronic pain in the aging population. Recent studies have identified several lncRNA-associated functions involved in the development of OA. Because age is a key risk factor for OA, we investigated the differential expression of age-related lncRNAs in each stage of OA.

**Methods:**

Two gene expression profiles were downloaded from the GEO database and differentially expressed genes (DEGs) were identified across each of the different developmental stages of OA. Next, gene ontology (GO) functional and Kyoto Encyclopedia of Genes and Genomes (KEGG) pathway analyses were performed to annotate the function of the DEGs. Finally, a lncRNA-targeted DEG network was used to identify hub-lncRNAs.

**Results:**

A total of 174 age-related DEGs were identified. GO analyses confirmed that age-related degradation was strongly associated with cell adhesion, endodermal cell differentiation and collagen fibril organization. Significantly enriched KEGG pathways associated with these DEGs included the PI3K–Akt signaling pathway, focal adhesion, and ECM–receptor interaction. Further analyses via a protein–protein interaction (PPI) network identified two hub lncRNAs, CRNDE and LINC00152, involved in the process of age-related degeneration of articular cartilage. Our findings suggest that lncRNAs may play active roles in the development of OA. Investigation of the gene expression profiles in different development stages may supply a new target for OA treatment.

## Introduction

Osteoarthritis (OA) is a common chronic joint disease in elderly individuals, with weight-bearing joints such as the knees, hips, and the spine being the most commonly affected. Between 2011 and 2012, the prevalence of symptomatic knee OA in China was 8.1% (Tang et al., 2016). OA progresses slowly over time, eventually resulting in joint pain, stiffness, and dysfunction (Hunter & Felson, 2006). With the growing number of patients suffering from OA, the treatment costs of OA are expected to present a huge economic burden to both individuals and health systems worldwide. While the exact mechanisms underlying OA remain poorly understood, numerous risk factors have been identified, including age, obesity, sex, genetic predisposition, and joint injury (Loeser, 2017; Silverwood et al., 2015). Among these factors, age is one of the most important critical influences on the degeneration of articular cartilage. Previous studies have demonstrated that the prevalence of radiographic knee OA increases with age (Shane Anderson & Loeser, 2010). Aging-related changes in joint tissues include accumulation of senescent secretory phenotype cells, increased expressions of catabolic factors, a gradual loss of extracellular cartilage matrix, and oxidative stress (Campisi & d’Addadi Fagagna, 2007; Ding et al., 2005; Henrotin, Bruckner & Pujol, 2003; Wu et al., 2002). Moreover, age-related increases in mesenchymal stromal cells in the bone marrow are also thought to regulate of osteoblast formation and bone remodeling (Ganguly et al., 2017).

Long non-coding RNAs (lncRNAs), defined as non-coding capacity RNAs >200 bp in length, play an important role in transcriptional regulation (Ponting, Oliver & Reik, 2009). Recent studies have identified several lncRNA-associated functions involved in the development of human diseases, including several forms of cancer, cardiovascular disease, diabetes, and osteoporosis (Cao et al., 2013; Hao et al., 2015; Huang, 2018; Leti & DiStefano, 2017; Wang et al., 2018b), as well as OA. Using DNA microarrays and bioinformatic approaches, Ming et al. identified a number of lncRNAs that are differentially expressed in OA cartilage compared to normal samples (Fu et al., 2015). Similarly, Xiang et al. found that synovium samples obtained from OA patients showed differential expression of numerous mRNAs and lncRNAs (Xiang et al., 2018). In addition, an increasing number of lncRNAs have been implicated in chondrocytes proliferation and cartilage metabolism. For example, increased expression of lncRNA-ZFAS1 was shown to induce the proliferation and migration of chondrocytes (Ye et al., 2018), while increased expression of lncRNA-HOTAIR contributes to chondrocytes apoptosis and matrix degradation (Hu et al., 2018). Finally, lncRNA-CIR acts as a sponge for miRNA-27b, resulting in increased extracellular matrix degradation via its effects on MMP13 expression (Li et al., 2017c). Taken together, these data suggest that lncRNAs play key roles in the pathogenesis of OA.

Because OA is an age-related chronic disease, morphological and molecular biological characters may appear differently in different stages. Here, to explore differential expression of age-related lncRNAs in each stage of OA, we examined two previously published OA gene datasets available in the Gene Expression Omnibus (GEO) database. Differential expression of lncRNAs across different developmental stages was examined by comprehensive bioinformatic analyses. Then the differentially expressed lncRNAs were correlated with other DEGs and potential diseases based on data obtained from the RAID and MNDR databases. Moreover, Gene Ontology (GO) and Kyoto Encyclopedia of Genes and Genomes (KEGG) pathway enrichment analyses were also performed, and a protein–protein interaction (PPI) network was used to screen for crucial genes and lncRNAs.

## Materials and Methods

### Expression profile dataset

**Figure 1:**
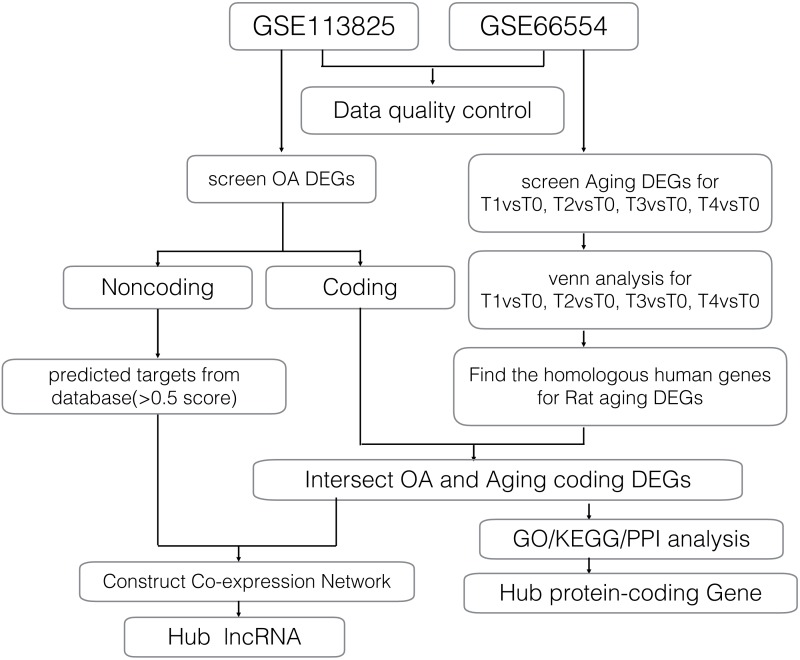
Workflow of the bioinformatics analyses.

Gene expression profiles for age-related degeneration in rat knee articular cartilage at different development stages (GSE66554) were obtained from the GEO database and analyzed alongside a second gene expression dataset (GSE113825) which investigated lncRNA and mRNA expression profiles of human knee OA. A detailed workflow of the data analyses is shown in Fig. 1. The quality of gene expression data was analyzed and visualized using the ggplot2 package of R software for each sample.

### Differential gene expression analyses

In GSE66554, the authors grouped rats as newborn (T0), youth (T1), adult (T2), early-stage elderly (T3), and later-stage elderly (T4). To identify age-related DEGs, we compared the different stages of rats with T0. We also identify DEGs between RNA-seq data of normal human cartilage samples and OA samples in GSE113825. The Linear Models for Microarray data (LIMMA) (limma) package, which includes lmFit, eBayes, and topTable functions, was used for pairwise comparison of DEGs (Ritchie et al., 2015). *P* < 0.05 and abs(log_2)_fold change (FC) >1 were used as the cut-off criteria.

### GO annotation and KEGG pathway enrichment analyses of DEGs

The R package bioMart was used to transform rats genes into homologous human genes. Then we narrowed down the protein coding DEGs by calculating intersection between these homologous human genes and OA DEGs (from GSE113825) to identify age-related OA DEGs. GO annotation, a classic method used to describe subcellular location, molecular function, and the biological attributes of DEGs, was applied (Ashburner et al., 2000). KEGG, a collection of databases summarizing genomes, biological pathways, and health information, was used to clarify the potential role of the DEGs (Kanehisa & Goto, 2000). GO functional analyses encompassing biological processes (BP), cell components (CC), and molecular functions (MF) was performed using the Database for Annotation, Visualization and Integrated Discovery (DAVID; ver. 6.8), with *P* < 0.05 used as a cutoff.

### PPI network construction and hub gene identification

The Search Tool for the Retrieval of Interacting Genes (STRING, http://string-db.org) was used to create a PPI network for the DEGs (Szklarczyk et al., 2015), which was visualized using Cytoscape (ver. 3.5.1). Genes served as “nodes” in the PPI network and the line segment between two nodes represented associated interactions. The color of the lines between genes indicates the degree of the interaction. The centiscape plugin was used to determine the degree of connectivity for each node in the PPI network. Genes with a degree >5 were defined as hub genes. The differentially expressed lncRNA-mRNA interaction network was built and displayed using Cytoscape. lncRNAs interacting with the greatest number of hub genes (mRNA) in the network were defined as hub lncRNAs.

### Patient samples preparation and qRT-PCR validation

This study was approved by the Ethics Committee of the 2nd Affiliated Hospital, School of medicine, Zhejiang University, Hangzhou, China (No.2018-043) and written informed consent was obtained for each participant. Femoral heads were collected from eight osteoarthritis patients and eight femoral neck fracture patients who underwent total hip arthroplasty. Then the cartilages obtained from the femoral head (nearly 1 cm*1 cm) were preserved in liquid nitrogen until use. Total RNA was extracted using TRIzol reagent (Thermo Fisher Scientific, Inc., Waltham, MA, USA) according to the manufacturer’s instructions. And 1µg RNA was reverse-transcribed using the cDNA synthesis kit (Thermo Fisher Scientific, Inc., Waltham, MA, USA). RT-PCR was performed using the SYBR Premix Ex Taq II (Takara Biotechnology, Dalian, China) system on the following steps: 45 cycles of 95 ° C for 15 s and 60 ° C for 30 s. The sequences of the primers used in the reaction are listed in Table 1. Relative gene expression was calculated using the 2^−ΔΔ*Ct*^ method. 18S served as the internal control gene.

**Table 1:** Real-Time PCR Primers.

ID	Primer Sequences(5′to 3′)
CRNDE	CTCTAGTCGTGTCCCCTCGT
	CTAGCCCACGGGACGTCTG
LINC00152	TGGCACAGTCTTTTCTCTACTCA
	GTCAAGAGGTTTCCAGGGGC
18S	CCTGAGAAACGGCTACCACA
	ACCAGACTTGCCCTCCAATG

### Statistical analysis

All data are presented as means ± standard deviation (SD). All experiments were performed at least three times. Student’s *t*-test was performed using SPSS (ver. 19.0; IBM Corp., Armonk, NY, USA) and GraphPad Prism 7 (GraphPad Software Inc., La Jolla, CA, USA) software. For all analyses, a *p* value <0.05 was considered to indicate statistical significance.

## Results

### Data distribution analyses and DEG screening

**Figure 2:**
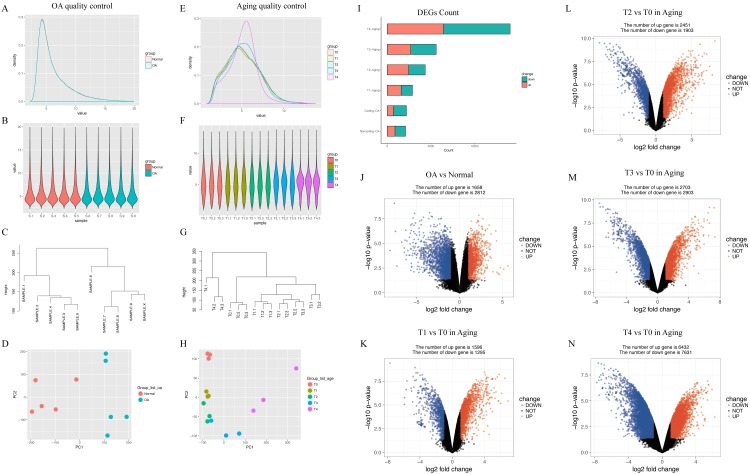
Distribution analyses of gene expression. (A) Distribution of gene expression levels in GSE113825. The x-axis indicates the log_2_ value (fragments per kilobase of transcript, per million fragments sequenced [FPKM]) and the y-axis shows the proportion of genes. (B) Distribution of genes expression for each sample in GSE113825. Individual samples are shown on the x-axis, with gene value distribution plotted on the y-axis. (C) Cluster analyses for all samples in GSE113825. (D) Principle component analyses of two groups in GSE113825, plotted as the first and second principal components. Dots represent the principal component value of each sample. (E–H) Represent the distribution analyses for GSE66554. (I) Number of differentially expressed genes (DEGs) identified in each group. (J–N) Volcano plots displaying pairs of expressed genes, where the x- and y-axis represent the log-transformed threshold values. Red dots indicate all significantly upregulated genes and blue dots indicate all significantly downregulated genes that passed the screening threshold. Black dots represent nonsignificant genes.

In GSE113825, a total of 95,722 genes were detected in each sample. The expression values (ranging from 0 to 20 log_2_ [fragments per kilobase of transcript, per million fragments sequenced, FPKM]) and the distributions were similar between normal group and OA group (Fig. 2A). Principal component and h-cluster analyses showed that samples were easily grouped into different groups. As for GSE66554, a total of 36685 genes were detected in each sample, with the values and distributions of these genes similar across the five groups (Fig. 2B). Based on these assessments, further bioinformatics analyses could be performed based on the available data.

Following data processing using limma, we identified 4470 DEGs (1658 upregulated, 2812 downregulated) in human normal vs. OA, including 2891 DEGs (1596 upregulated, 1295 downregulated) in rat T0 vs. rat T1, 4354 DEGs (2451 upregulated, 1903 downregulated) in rat T0 vs. rat T2, 5606 DEGs (2703 upregulated, 2903 downregulated) in rat T0 vs. rat T3, and 14063 DEGs (6432 upregulated, 7631 downregulated) in rat T0 vs. rat T4 (Fig. 2C).

### Intersection between the predicted targets of lncRNAs and DEGs

**Figure 3:**
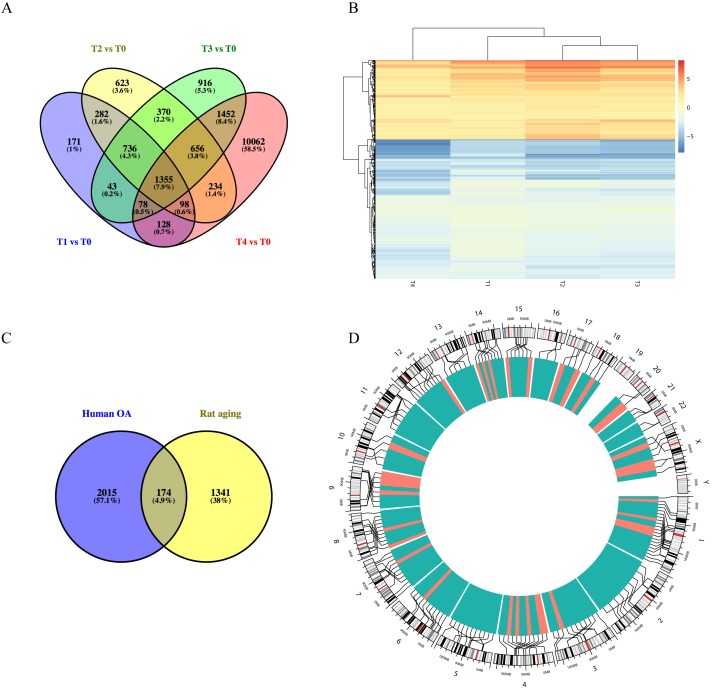
Functional analyses of differentially expressed long non-coding RNA (lncRNA)-targeted DEGs. (A) Venn diagram of DEGs across different growth stages in GSE66554. (B) Hierarchical clustering analyses of DEGs common to all four groups. (C) Venn diagram demonstrating the overlap between the OA aging groups. A total of 174 DEGs were identified as age-related genes, including 33 upregulated and 141 downregulated genes. (D) Circle graph showing the detailed location of 174 DEGs across the human genome. Chromosome locations are marked by lines, upregulated genes shown in red and downregulated genes shown in green. Chromosomes 1, 13, and 4 were most common locations for age-related DEGs in OA.

Using the GSE66554 rat OA dataset, we identified a total of 1355 DEGs common across each of the different stages (Fig. 3A). Hierarchical clustering of the identified DEGs is displayed as a heatmap in Fig. 3B. Next, homologous human genes were identified for each of the rat DEGs and compared against DEGs identified in the human OA dataset (Fig. 3C), resulting in an overlap of 174 age-related OA genes, including 33 upregulated and 141 downregulated genes (Table S1). Alignment of these to the human genome is shown in Fig. 3D, as intuitively, green means go and red means stop/downregulated.

### GO annotation and KEGG pathway enrichment analyses

**Figure 4:**
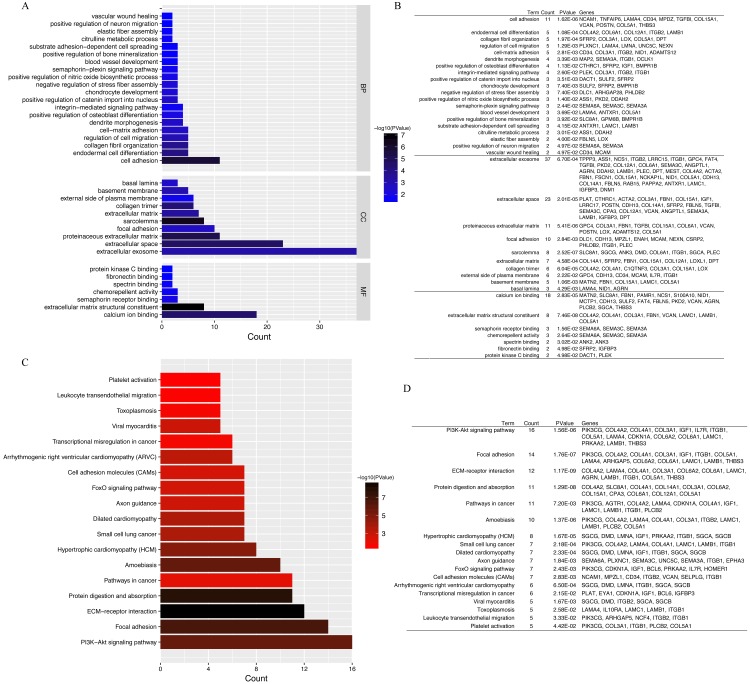
GO functional classification and KEGG pathways enrichment in DEGs. (A) Histogram of Gene Ontology (GO) functional classification of DEGs. The x-axis represents the number of DEGs, with individual GO terms plotted on the y-axis. All GO terms were grouped into three categories: biological processes, cellular components, and molecular functions. The graph displays only significantly enriched GO terms (*P* < 0.05), with darker blue indicating greater significance. (B) Top BP, CC, and MF terms and their corresponding genes in GO functional enrichment analyses. (C) Histogram of Kyoto Encyclopedia of Genes and Genomes (KEGG) pathways enrichment in DEGs. The x-axis represents the number of DEGs annotated in a pathway, with individual KEGG terms shown on the y-axis. The graph displays only significantly enriched KEGG terms (*P* < 0.05), with darker red indicating greater significance. (D) Individual KEGG terms are shown for each group.

GO annotation and KEGG pathway enrichment analyses were performed to better understand the functional significance of the DEGs. Biological processes (BP) were significantly enriched for cell adhesion, endodermal cell differentiation, collagen fibril organization, regulation of cell migration, and cell–matrix adhesion (Fig. 4A). Significant cellular component (CC) terms revealed enrichment in extracellular exosome, extracellular space, proteinaceous extracellular matrix, focal adhesion, and sarcolemma. Finally, the top five molecular functions (MF) identified were calcium ion binding, extracellular matrix structural constituent, semaphorin receptor binding, chemorepellent activity, and spectrin binding. All significantly enriched entries are shown in a histogram in Fig. 4B. KEGG analyses of the DEGs revealed enrichment for 18 terms (Fig. 4C). The top five significantly enriched KEGG pathways were the PI3K–Akt signaling pathway, focal adhesion, ECM–receptor interaction, protein digestion and absorption, and pathways in cancer. Detailed information regarding each of the DEGs involved in the BP analyses and KEGG pathway are listed in Fig. 4.

### PPI network construction and hub lncRNAs identification

**Figure 5:**
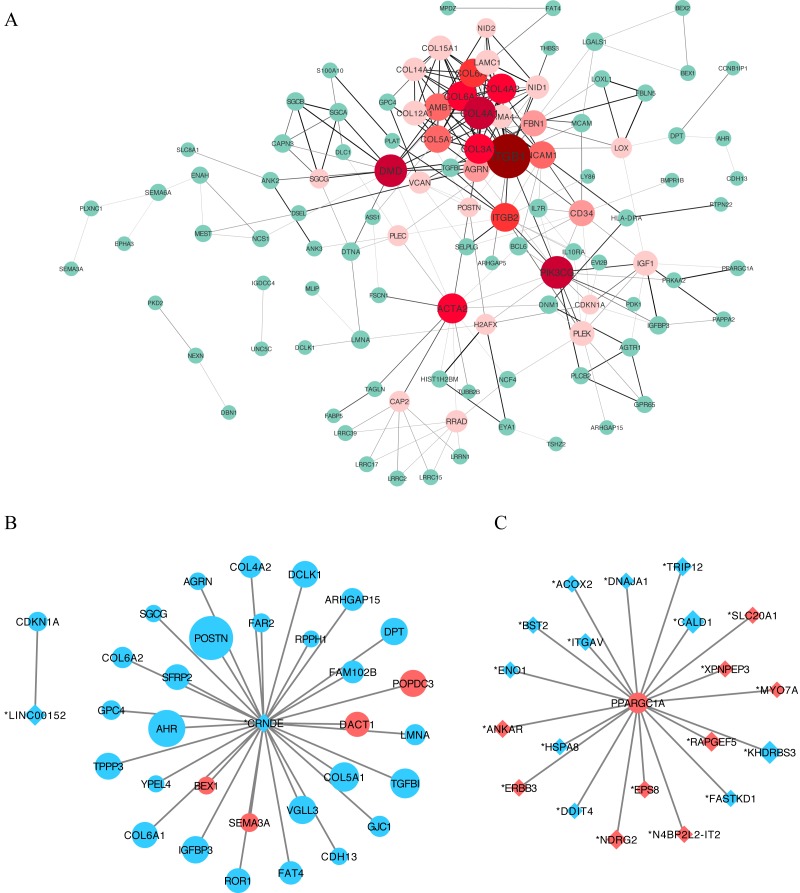
Protein–protein interaction (PPI) network construction. (A) A PPI network was used to screen for lncRNA-targeted DEGs. Genes marked in red represent hub genes with a degree of interaction ≥ 5. The darker the connection line, the greater the confidence score. A total of 34 DEGs were identified as age-related hub genes. (B) lncRNA-mRNA association network. Circles indicate mRNAs, with diamonds used to indicate non-protein-coding RNAs. A larger node indicates a larger log_2FC value. Upregulated genes are shown in red and downregulated genes are shown in blue. Here we identified two hub-lncRNAs, CRNDE and LINC00152 and constructed their lncRNA-mRNA networks in graph B. (C) PPARGC1A-lncRNA network. PPARGC1A was a novel DEG which exhibits a close relationship with differently identified age-related lncRNAs.

We constructed a putative PPI network map for the overlapping DEGs using the STRING database, which was visualized with Cytoscape. Excluding the DEGs distributed on the edge of the PPI network, the remaining 110 central DEGs were evaluated using the centiscape plugin. After degree calculation, a total of 34 hub genes were colored red in the PPI network (Fig. 5A and Table 2). Using this same approach, we also constructed a lncRNA–gene co-expression network. Each hub lncRNA was determined by calculating its downstream hub genes whose count should be ranked in the top three (minimum of two genes). A total of 23 lncRNAs were screened and are presented in Table 3. From this analysis, the lncRNAs CRNDE (target DEGs: COL4A2, COL6A2, COL6A1, COL5A1, AGRN, POSTN and SGCG) and LINC00152 (target DEG: CDKN1A) stood out. We also identified an important hub gene, PPARGC1A, which exhibited tight connections with other lncRNAs (Fig. 5C).

### qRT-PCR validation

**Figure 6:**
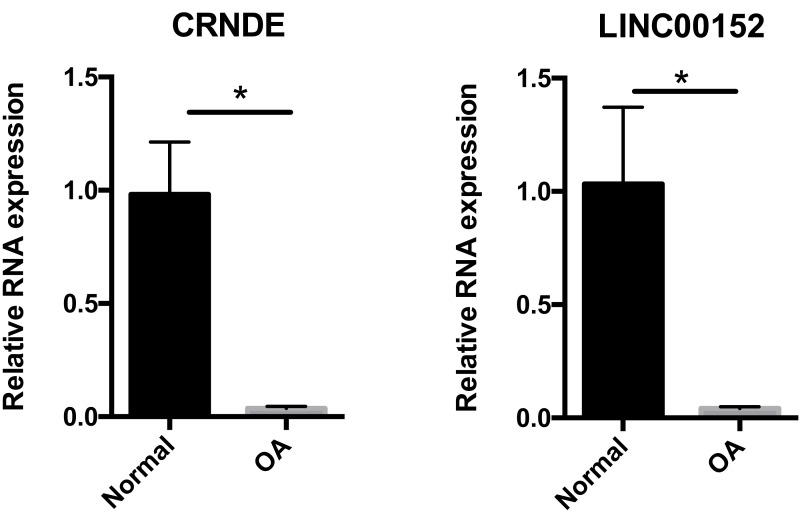
Relative expression of CRNDE (A) and LINC00152 (B) in human samples. Relative expression of CRNDE (A) and LINC00152 (B) in human samples, as determined by RT-PCR. Each column represents the mean ± SD. The OA group exhibited lower-level expression of CRNDE and LINC00152 compared with the normal group. OA samples were obtained from eight osteoarthritis patients who underwent total hip arthroplasty. Normal samples were obtained from eight femoral neck fracture patients who underwent total hip arthroplasty. *p < 0.05 vs. normal group.

To confirm the results of our bioinformatic analysis, we examined the expression of CRNDE and LNC00152 by qRT-PCR in 16 cartilage samples. According to Fig. 6, the transcriptional levels of CRNDE and LNC00152 were significantly decreased in OA group compared with the normal group. These results were consistent with our previous integrative analysis listed in Table 3 and showed the same trends of these hub lncRNAs.

**Table 2:** Hub-genes identified by centiscape (degree ≥ 5) among 174 DEGs.

Name	logFC	AveExpr	P.Value	Transcript_id	Degree
ITGB1	−1.75	16.20	5.68E–04	NM_002211	24
COL4A1	−2.08	6.32	1.06E–05	NM_001845	15
DMD	−2.54	5.21	1.16E–04	NM_000109	15
PIK3CG	−2.76	5.00	2.46E–03	NM_002649	15
ACTA2	−3.98	8.71	2.63E–05	NM_001141945	13
COL3A1	−2.17	14.51	9.08E–03	NM_000090	13
COL4A2	−2.39	5.86	1.27E–06	NM_001846	13
COL6A2	−2.16	7.79	5.55E–03	NM_001849	13
COL6A1	−3.24	12.98	2.16E–03	NM_001848	12
ITGB2	−1.57	5.28	2.96E–02	NM_000211	12
COL5A1	−3.98	12.58	4.02E–03	NM_000093	11
LAMB1	−4.11	7.02	6.24E–05	NM_002291	11
NCAM1	−2.70	9.34	8.57E–03	NM_000615	11
AGRN	−1.29	5.82	3.74E–05	NM_198576	10
CD34	−2.22	8.14	8.45E–03	NM_001025109	10
FBN1	−2.32	12.40	2.56E–05	NM_000138	10
COL12A1	−2.64	14.08	4.18E–05	NM_004370	9
COL15A1	−3.08	8.66	7.14E–03	NM_001855	9
IGF1	−2.17	8.53	2.33E–04	NM_000618	9
LAMC1	−1.14	5.81	4.06E–03	NM_002293	9
PLEK	−3.59	7.30	2.46E–04	NM_002664	9
COL14A1	−4.13	12.30	7.96E–04	NM_021110	8
LAMA4	−1.82	7.39	8.45E–05	NM_001105206	8
NID1	−4.19	9.38	2.22E–08	NM_002508	8
RRAD	−1.19	4.96	3.31E–03	NM_001128850	7
VCAN	−4.09	10.96	2.68E–04	NM_001126336	7
CAP2	−1.17	6.67	2.07E–03	NM_006366	6
H2AFX	−1.19	6.96	2.48E–04	NM_002105	6
LOX	−2.88	11.46	1.92E–03	NM_001178102	6
PLEC	−1.05	8.23	4.22E–03	NM_000445	6
CDKN1A	−1.73	7.04	6.11E–03	NM_000389	5
NID2	−3.56	9.41	3.20E–05	NM_007361	5
POSTN	−7.29	10.68	1.97E–03	NM_001135934	5
SGCG	−1.26	4.39	3.62E–02	NM_000231	5

**Table 3:** All differentially expressed lncRNAs upstream of DEGs.

Gene symbol	logFC	AveExpr	P.Value	change
ACOX2	−1.29	5.88	1.83E–05	DOWN
ANKAR	1.44	9.79	6.63E–05	UP
BST2	−1.26	6.01	8.68E–04	DOWN
CALD1	−2.03	11.76	8.24E–05	DOWN
CRNDE	−1.62	6.96	5.43E–06	DOWN
DDIT4	−1.08	6.48	6.00E–03	DOWN
DNAJA1	−1.18	10.99	1.41E–04	DOWN
ENO1	−1.24	12.23	3.24E–02	DOWN
EPS8	1.39	10.73	2.24E–04	UP
ERBB3	1.35	6.31	1.90E–03	UP
FASTKD1	−1.27	10.04	2.31E–03	DOWN
HSPA8	−1.20	14.94	7.94E–05	DOWN
ITGAV	−1.35	9.69	6.15E–04	DOWN
KHDRBS3	−2.58	7.15	4.01E–04	DOWN
LINC00152	−2.20	8.75	2.19E–04	DOWN
MYO7A	1.09	6.56	4.67E–03	UP
N4BP2L2-IT2	1.27	11.17	1.64E–04	UP
NDRG2	1.83	9.05	9.92E–03	UP
RAPGEF5	1.88	5.55	2.01E–05	UP
SLC20A1	1.23	11.19	1.77E–02	UP
TMEM161B-AS1	1.02	7.91	1.70E–05	UP
TRIP12	−1.67	10.53	1.84E–04	DOWN
XPNPEP3	1.22	7.23	1.04E–03	UP

## Discussion

LncRNAs have emerged as critical modulators of transcriptional, post-transcriptional, and epigenetic gene regulation, with emerging evidence suggesting a relationship between lncRNAs and OA. With the development of high-throughput sequencing technology, an increasing number of RNA-sequencing projects have been performed, identifying key functional mRNAs, miRNAs, lncRNAs, and circRNAs in the development of OA. Xing et al. (2014) reported six lncRNAs, including HOTAIR, GAS5, PMS2L2, RP11–445H22.4, H19, and CTD-2574D22.4, which were differently expressed in OA samples in microarray analyses (Xing et al., 2014). Subsequent studies have since described lncRNAs expression patterns in human osteoarthritic cartilage using a combination of DNA microarray and bioinformatic analyses (Fu et al., 2015). Additional lncRNAs including PACER, CILinc01, and CILinc02, have also been reported in human hip OA chondrocytes, and were significantly associated with the OA inflammatory response (Pearson et al., 2016). Similarly, when comparing the expression of lncRNAs in OA cartilage of variable severity, four hub lncRNAs, SNHG5, ZFAS1, GAS5, and DANCR, were identified as key functional mediators in OA pathogenesis (Xiao et al., 2018).

Although many bioinformatics analyses have revealed differences in gene expression patterns in OA samples, few have examined what, if any, age-related lncRNAs are involved in this process. Therefore, a detailed database of lncRNA observed in rat knee articular cartilage at different developmental stages was used in our study. By comparing differential gene expression in five stages, newborn (T0), youth (T1), adult (T2), early-stage elderly (T3), and later-stage elderly (T4), a set of age-related DEGs were identified. Next, we identified the homologous human genes for each of our age-related rat DEGs, which then were compared against a separate set of DEGs extracted from a human OA lncRNAs expression database. Our results broadly confirmed dysregulated gene in age-related cartilage, including ITGB1, COL4A1, DMD, PIK3CG, ACTA2, COL3A1, COL4A2, COL6A2, and COL6A1. We also identified two hub lncRNAs, CRNDE and LINC00152, which were not identified in previous studies.

**Figure 7:**
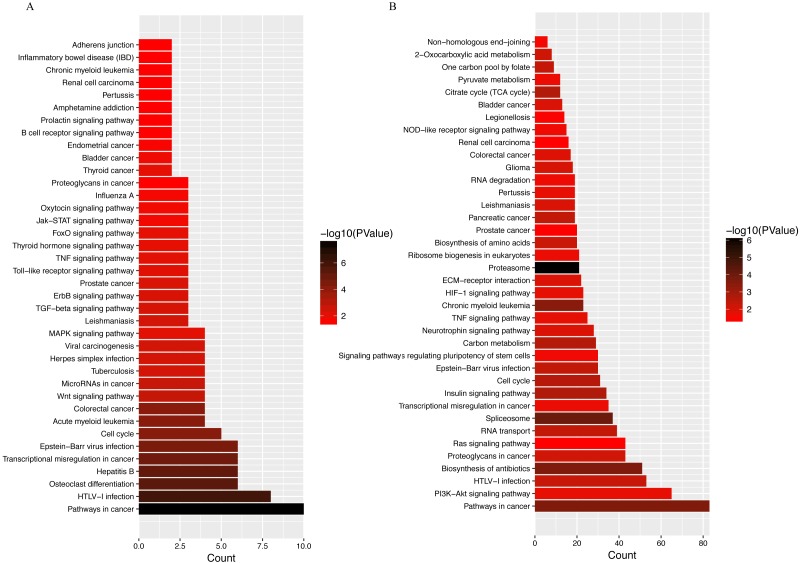
KEGG analyses of (A) LINC00152 and (B) CRNDE. The x-axis represents the number of predicted target gene, with individual KEGG terms shown on the y-axis.

LINC00152 is an 828 bp lncRNA located on chromosome 2p11.2. Previous studies have found that LINC00152 plays an oncogenic role in the development of a wide range of tumor types (Chen et al., 2018a; Chen et al., 2018c). Enhanced LINC00152 expression has also been found to be a potential prognostic biomarker in patients with lung and colorectal cancers (Chen et al., 2018b; Li et al., 2017b). LINC00152 also acts as a sponge for various miRNAs, as shown in a recent report describing the interaction between LINC00152 and miR-139 in colorectal cancer cells (Bian et al., 2017). This observation is important, as emerging evidence has shown increased expression of miR-139 in OA cartilage, with increased miR-139 expression resulting in higher expression of IL-6 and chondrocyte apoptosis (Hu et al., 2016; Makki & Haqqi, 2015). Furthermore, KEGG analyses of LINC00152 (Fig. 7A) revealed strong associations between osteoclast differentiation, cell cycle, and Wnt signaling pathways and LINC00152 in OA. Given these observations, the LINC00152/miRNA axis will likely become a major focus of future OA studies.

Another hub lncRNA identified in our study, CRNDE, is a 1910 nt cancer-secreted lncRNA transcribed by human chromosome 16 (16q12.2). This lncRNA has been observed in a variety of cancers, including osteosarcoma, colorectal, cervical, and gastric cancers (Ding et al., 2017; Hu et al., 2017; Li et al., 2018; Meng et al., 2017). Huan et al. (2017) reported an important role for CRNDE in the pathophysiology of human breast cancer. CRNDE was shown to modulate the Wnt/β-catenin signaling pathway by repressing miR-136 expression via the miRNA sponge mechanism, resulting in significant difference in proliferation, migration, and invasion of breast cancer cells. Coincidentally, another group demonstrated that miR-136 plays an important role in the regulation of chondrogenesis in human adipose-derived stem cells (Zhang et al., 2012). Further research revealed that miR-136 bound to the 3′-UTR of MMP13, a key catabolic enzyme involved in the degradation of ECM (Li et al., 2017a). By competitively binding with miR-136, circRNA-CER regulates MMP13 expression, further promoting chondrocyte ECM degradation (Liu et al., 2016). Beyond miR136, decades of research have shown that CRNDE modulates miR-384 activity in a number of tumor cell types (Chen et al., 2016; Zheng et al., 2016). Recently, miR-384–5p was shown to induce OA by regulating the expression of SOX9 and downregulating activity of the NF-κB signaling pathway (Zhang et al., 2018). Taken together, these data suggest that CRNDE can act as a miRNA sponge, competing for miRNA binding with protein-coding transcripts, although the exact mechanism of lncRNA CRNDE-mediated regulation of OA pathogenesis remains to be investigated. Other potential miRNAs related to these two hub lncRNAs have been curated from databases (RAID 2.0, starBASE v2.0, miRTarBase), and are listed in Table 4. Targets suggested by KEGG analyses suggest that the PI3K–Akt signaling pathway, proteoglycan metabolism, and the Ras signaling pathway are promising targets (Fig. 7B).

**Table 4:** Potential functional miRNAs associated with hub lncRNAs.

predicted miRNAs for LINC00152		predicted miRNAs for CRNDE	
hsa-miR-106a-5p		hsa-miR-136-5p	
hsa-miR-125a-3p		hsa-miR-384	
hsa-miR-129-5p		hsa-miR-145-5p	
hsa-miR-136-5p			
hsa-miR-1-3p			
hsa-miR-149-5p			
hsa-miR-185-5p			
hsa-miR-193a-3p			
hsa-miR-193b-3p			
hsa-miR-206			
hsa-miR-24-3p			
hsa-miR-320a			
hsa-miR-320b			
hsa-miR-320c			
hsa-miR-320d			
hsa-miR-376a-3p			
hsa-miR-376b-3p			
hsa-miR-376c-3p			
hsa-miR-383-5p			
hsa-miR-485-5p			
hsa-miR-503-5p			
hsa-miR-613			
hsa-miR-873-5p			

Outside of lncRNAs, the PPI network identified PPARGC1A as a novel mediator of OA pathogenesis due to its close relationship with various age-related lncRNAs. PPARGC1A, also known as human-accelerated region 20, encodes the peroxisome proliferator-activated receptor gamma coactivator 1-alpha (PGC-1α). PGC-1 α is widely viewed as a master regulator of mitochondrial biogenesis (Liang & Ward, 2006). Reduced expression of PGC-1α has been observed in knee cartilage of aged mice, where it may attenuate oxidative stress and cartilage erosion in OA (Zhao et al., 2014). Activation of PGC-1α expression significantly enhances mitochondrial biogenesis and inhibits oxidative phosphorylation in OA chondrocytes. Similarly, the AMP-activated protein kinase/PGC-1 signaling pathway is an effective target for OA treatment in a rat model (Wang et al., 2018a).

## Conclusions

The current study identified a series of DEGs and lncRNAs in each developmental stage of articular cartilage. Using a series of bioinformatics analyses, two pivotal lncRNAs, CRNDE and LINC00152, were identified, which are strongly associated with age-related cartilage degradation. Further functional analyses suggest a potential mechanism through which these hub lncRNAs mediate OA pathogenesis. Our results highlight the important role of lncRNAs in the pathogenesis of OA, although more research is necessary to confirm our findings.

##  Supplemental Information

10.7717/peerj.7024/supp-1 Table S1 DEGs identified in the OA and aging groups

## Additional Information and Declarations

### Data Availability

The following information was supplied regarding data availability: Data is available in the GEO database, accession numbers: GSE66554 and GSE113825.
