# Supplementary material for: Identify CRNDE and LINC00152 as the key lncRNAs in age-related degeneration of articular cartilage through comprehensive and integrative analysis
Source: PeerJ. 2019 May 28;7:e7024. doi: 10.7717/peerj.7024 (PMC6544125; doi:10.7717/peerj.7024)
Supplement: Table S1 [file peerj-07-7024-s001.docx]

Table S1 174 DEGs in both OA group and aging group

| Gene symbol | p-value | Change | logFC | Chr | Start | End |
| --- | --- | --- | --- | --- | --- | --- |
| AGRN | 3.74E-05 | DOWN | -1.29 | chr1 | 1020123 | 1056119 |
| PRKAA2 | 1.99E-04 | UP | 1.25 | chr1 | 56645317 | 56715335 |
| ROR1 | 2.90E-06 | DOWN | -2.94 | chr1 | 63774019 | 64181498 |
| NEXN | 1.15E-05 | DOWN | -2.15 | chr1 | 77888513 | 77943895 |
| FAM102B | 7.41E-05 | DOWN | -2.48 | chr1 | 108560089 | 108644900 |
| LMNA | 1.49E-03 | DOWN | -2.11 | chr1 | 156082573 | 156140089 |
| MPZL1 | 7.00E-03 | DOWN | -1.11 | chr1 | 167721192 | 167791919 |
| PAPPA2 | 3.54E-03 | UP | 2.49 | chr1 | 176463171 | 176845599 |
| LAMC1 | 4.06E-03 | DOWN | -1.14 | chr1 | 183023460 | 183145592 |
| DYRK3 | 5.45E-03 | DOWN | -1.02 | chr1 | 206635536 | 206684419 |
| LRRC39 | 1.88E-03 | UP | 1.46 | chr1 | 100148448 | 100178273 |
| PTPN22 | 1.56E-04 | UP | 1.82 | chr1 | 113813811 | 113871759 |
| TBX15 | 1.71E-04 | UP | 1.11 | chr1 | 118883043 | 118989556 |
| S100A10 | 3.58E-03 | DOWN | -2.25 | chr1 | 151982910 | 151994390 |
| THBS3 | 1.60E-02 | DOWN | -1.69 | chr1 | 155195588 | 155209178 |
| ARHGAP30 | 4.16E-02 | DOWN | -1.14 | chr1 | 161046942 | 161069970 |
| DPT | 5.13E-04 | DOWN | -3.50 | chr1 | 168695457 | 168729264 |
| ANGPTL1 | 1.25E-03 | DOWN | -1.53 | chr1 | 178849535 | 178871080 |
| CD34 | 8.45E-03 | DOWN | -2.22 | chr1 | 207884249 | 207911402 |
| ENAH | 4.05E-03 | DOWN | -1.37 | chr1 | 225486832 | 225653143 |
| NID1 | 2.22E-08 | DOWN | -4.19 | chr1 | 235975830 | 236065181 |
| PLEK | 2.46E-04 | DOWN | -3.59 | chr2 | 68365173 | 68397453 |
| ANTXR1 | 3.75E-03 | DOWN | -1.44 | chr2 | 69013144 | 69249327 |
| ARHGAP15 | 1.60E-02 | DOWN | -2.49 | chr2 | 143091362 | 143768352 |
| TNFAIP6 | 5.56E-03 | DOWN | -5.68 | chr2 | 151357592 | 151380048 |
| PDK1 | 3.66E-02 | DOWN | -1.50 | chr2 | 172555373 | 172625095 |
| COL3A1 | 9.08E-03 | DOWN | -2.17 | chr2 | 188974320 | 189012746 |
| SPATS2L | 2.07E-04 | DOWN | -1.33 | chr2 | 200305881 | 200482263 |
| MAP2 | 6.07E-04 | DOWN | -2.08 | chr2 | 209424047 | 209734118 |
| SLC8A1 | 2.45E-03 | DOWN | -1.83 | chr2 | 40112146 | 40611053 |
| LRRN1 | 2.12E-02 | DOWN | -1.56 | chr3 | 3799437 | 3847703 |
| EPHA3 | 1.84E-02 | DOWN | -1.14 | chr3 | 89107524 | 89482134 |
| AGTR1 | 9.63E-04 | DOWN | -2.61 | chr3 | 148697784 | 148743008 |
| CPA3 | 1.82E-04 | DOWN | -4.18 | chr3 | 148865256 | 148897196 |
| CCR1 | 7.26E-05 | DOWN | -3.94 | chr3 | 46201709 | 46208396 |
| LRRC2 | 8.26E-03 | DOWN | -1.28 | chr3 | 46515388 | 46580099 |
| VGLL3 | 1.78E-05 | DOWN | -3.70 | chr3 | 86937969 | 86991119 |
| FILIP1L | 3.49E-05 | DOWN | -5.63 | chr3 | 99830141 | 100114513 |
| BCL6 | 3.93E-04 | UP | 1.82 | chr3 | 187721377 | 187745727 |
| LRRC15 | 7.42E-06 | DOWN | -6.72 | chr3 | 194355247 | 194369743 |
| PHLDB2 | 2.67E-04 | DOWN | -1.85 | chr3 | 111859180 | 111976517 |
| PARM1 | 8.49E-05 | UP | 2.22 | chr4 | 74933075 | 75050115 |
| PKD2 | 4.59E-05 | UP | 1.59 | chr4 | 88007647 | 88077779 |
| BMPR1B | 2.11E-03 | DOWN | -2.46 | chr4 | 94757968 | 95158450 |
| ANK2 | 3.22E-02 | DOWN | -1.02 | chr4 | 112818083 | 113388728 |
| MYOZ2 | 1.70E-05 | UP | 1.32 | chr4 | 119135784 | 119187789 |
| SPRY1 | 3.33E-05 | DOWN | -2.74 | chr4 | 123396795 | 123403755 |
| FAT4 | 1.62E-05 | DOWN | -2.63 | chr4 | 125316399 | 125492932 |
| PPARGC1A | 7.91E-08 | UP | 2.00 | chr4 | 23755041 | 23904089 |
| SGCB | 1.03E-03 | DOWN | -1.58 | chr4 | 52020695 | 52038482 |
| UNC5C | 1.19E-04 | UP | 2.05 | chr4 | 95162504 | 95549210 |
| ANKRD50 | 2.30E-02 | DOWN | -1.11 | chr4 | 124664049 | 124712732 |
| SFRP2 | 1.10E-04 | DOWN | -3.14 | chr4 | 153780590 | 153789120 |
| IL7R | 8.39E-03 | DOWN | -2.96 | chr5 | 35852695 | 35879603 |
| VCAN | 2.68E-04 | DOWN | -4.09 | chr5 | 83471465 | 83582303 |
| TGFBI | 6.48E-03 | DOWN | -4.08 | chr5 | 136028895 | 136063818 |
| ADAMTS12 | 1.89E-03 | DOWN | -1.95 | chr5 | 33523535 | 33892192 |
| LHFPL2 | 3.24E-05 | DOWN | -2.25 | chr5 | 78485215 | 78770021 |
| HOMER1 | 3.30E-04 | DOWN | -2.17 | chr5 | 79372636 | 79514217 |
| MCTP1 | 8.00E-05 | DOWN | -2.82 | chr5 | 94703741 | 95284575 |
| SEMA6A | 6.55E-04 | DOWN | -1.86 | chr5 | 116443555 | 116574934 |
| LOX | 1.92E-03 | DOWN | -2.88 | chr5 | 122063195 | 122078511 |
| DBN1 | 3.05E-04 | DOWN | -1.82 | chr5 | 177456608 | 177474401 |
| C1QTNF3 | 2.42E-02 | DOWN | -1.45 | chr5 | 34017858 | 34043832 |
| LY86 | 6.17E-04 | DOWN | -3.23 | chr6 | 6588108 | 6654983 |
| CAP2 | 2.07E-03 | DOWN | -1.17 | chr6 | 17393216 | 17557792 |
| HIST1H2BM | 1.34E-04 | DOWN | -2.88 | chr6 | 27815044 | 27815489 |
| HLA-DRA | 1.98E-04 | DOWN | -4.53 | chr6 | 32439842 | 32445049 |
| CDKN1A | 6.11E-03 | DOWN | -1.73 | chr6 | 36676460 | 36687339 |
| TUBB2B | 2.86E-02 | DOWN | -1.47 | chr6 | 3224261 | 3231730 |
| COL12A1 | 4.18E-05 | DOWN | -2.64 | chr6 | 75084326 | 75206051 |
| POPDC3 | 1.39E-07 | UP | 3.45 | chr6 | 105157900 | 105179995 |
| LAMA4 | 8.45E-05 | DOWN | -1.82 | chr6 | 112107931 | 112254939 |
| MLIP | 3.08E-02 | DOWN | -1.31 | chr6 | 54018916 | 54266280 |
| DDAH2 | 5.16E-04 | DOWN | -1.53 | chr6 | 31727038 | 31730263 |
| FSCN1 | 7.19E-03 | DOWN | -1.64 | chr7 | 5592805 | 5606656 |
| AHR | 9.16E-07 | DOWN | -5.72 | chr7 | 17298622 | 17346152 |
| LRRC17 | 4.27E-04 | DOWN | -2.04 | chr7 | 102912897 | 102945109 |
| PIK3CG | 2.46E-03 | DOWN | -2.76 | chr7 | 106865278 | 106907147 |
| MEST | 1.38E-05 | DOWN | -3.82 | chr7 | 130486175 | 130506297 |
| IGFBP3 | 9.38E-03 | DOWN | -3.53 | chr7 | 45912245 | 45921874 |
| SEMA3C | 6.39E-05 | DOWN | -4.64 | chr7 | 80742538 | 80922359 |
| SEMA3A | 6.27E-03 | UP | 1.58 | chr7 | 83958343 | 84492724 |
| LAMB1 | 6.24E-05 | DOWN | -4.11 | chr7 | 107923799 | 108003359 |
| PTN | 5.99E-04 | DOWN | -3.02 | chr7 | 137227341 | 137343865 |
| CLEC5A | 2.56E-02 | DOWN | -1.65 | chr7 | 141927357 | 141947007 |
| PIWIL2 | 8.36E-03 | UP | 1.01 | chr8 | 22275297 | 22357563 |
| FABP5 | 5.55E-05 | DOWN | -2.79 | chr8 | 81280363 | 81284777 |
| MATN2 | 2.19E-03 | DOWN | -2.88 | chr8 | 97868840 | 98036718 |
| CTHRC1 | 3.09E-02 | DOWN | -1.51 | chr8 | 103371515 | 103383004 |
| COL14A1 | 7.96E-04 | DOWN | -4.13 | chr8 | 120059780 | 120372036 |
| ERICH1 | 1.20E-04 | UP | 3.24 | chr8 | 614744 | 738106 |
| DLC1 | 2.46E-03 | DOWN | -1.40 | chr8 | 13083361 | 13515658 |
| EBF2 | 1.80E-03 | DOWN | -1.63 | chr8 | 25841730 | 26045397 |
| PLAT | 2.08E-02 | DOWN | -2.29 | chr8 | 42174718 | 42207724 |
| EYA1 | 2.27E-02 | DOWN | -1.81 | chr8 | 71197433 | 71362232 |
| PLEC | 4.22E-03 | DOWN | -1.05 | chr8 | 143915153 | 143976745 |
| COL15A1 | 7.14E-03 | DOWN | -3.08 | chr9 | 98943179 | 99070787 |
| DNM1 | 8.44E-03 | DOWN | -1.38 | chr9 | 128203379 | 128255248 |
| NCS1 | 1.01E-03 | DOWN | -1.42 | chr9 | 130172578 | 130237304 |
| ASS1 | 1.34E-03 | UP | 1.22 | chr9 | 130444707 | 130501274 |
| COL5A1 | 4.02E-03 | DOWN | -3.98 | chr9 | 134641774 | 134844842 |
| MPDZ | 4.54E-04 | UP | 1.15 | chr9 | 13105704 | 13279590 |
| NFIB | 3.44E-03 | UP | 1.35 | chr9 | 14081843 | 14398983 |
| CNTFR | 1.43E-03 | UP | 2.28 | chr9 | 34551432 | 34590140 |
| SLC16A2 | 7.13E-04 | DOWN | -1.27 | chrX | 74420803 | 74533917 |
| GPM6B | 3.48E-02 | UP | 1.51 | chrX | 13770943 | 13938712 |
| DMD | 1.16E-04 | DOWN | -2.54 | chrX | 31114691 | 33339609 |
| SLC38A5 | 1.32E-02 | DOWN | -1.37 | chrX | 48458537 | 48470256 |
| BEX1 | 5.49E-03 | UP | 1.37 | chrX | 103062651 | 103064240 |
| BEX2 | 8.87E-04 | UP | 2.59 | chrX | 103309346 | 103311046 |
| GPC4 | 2.96E-04 | DOWN | -1.76 | chrX | 133300103 | 133415490 |
| BICC1 | 6.59E-05 | DOWN | -1.35 | chr10 | 58513137 | 58831435 |
| PRTFDC1 | 1.10E-02 | DOWN | -1.14 | chr10 | 24848625 | 24952606 |
| ITGB1 | 5.68E-04 | DOWN | -1.75 | chr10 | 32900318 | 33005792 |
| ANK3 | 4.72E-02 | UP | 1.96 | chr10 | 60026298 | 60733526 |
| ACTA2 | 2.63E-05 | DOWN | -3.98 | chr10 | 88935074 | 88991390 |
| NCAM1 | 8.57E-03 | DOWN | -2.70 | chr11 | 112961247 | 113278436 |
| TAGLN | 4.23E-03 | DOWN | -2.04 | chr11 | 117199321 | 117204807 |
| IL10RA | 1.29E-03 | DOWN | -2.82 | chr11 | 117986348 | 118001484 |
| PAMR1 | 1.71E-03 | DOWN | -1.30 | chr11 | 35431823 | 35530300 |
| YPEL4 | 1.44E-04 | DOWN | -1.20 | chr11 | 57645087 | 57649944 |
| H2AFX | 2.48E-04 | DOWN | -1.19 | chr11 | 119093854 | 119095467 |
| MCAM | 6.46E-04 | DOWN | -3.31 | chr11 | 119308524 | 119317130 |
| EMP1 | 1.16E-04 | DOWN | -1.90 | chr12 | 13196668 | 13216774 |
| MGST1 | 3.05E-03 | DOWN | -2.34 | chr12 | 16347142 | 16377192 |
| FAR2 | 2.75E-03 | DOWN | -1.63 | chr12 | 29149200 | 29334073 |
| NCKAP1L | 1.04E-03 | DOWN | -2.56 | chr12 | 54497711 | 54543942 |
| PLXNC1 | 1.24E-02 | DOWN | -1.28 | chr12 | 94148723 | 94307675 |
| HSPB8 | 2.42E-02 | DOWN | -1.80 | chr12 | 119178642 | 119194746 |
| CSRP2 | 1.39E-04 | DOWN | -3.93 | chr12 | 76858715 | 76879060 |
| IGF1 | 2.33E-04 | DOWN | -2.17 | chr12 | 102395867 | 102480645 |
| SELPLG | 2.82E-05 | DOWN | -2.69 | chr12 | 108621904 | 108633959 |
| CLEC2D | 2.59E-04 | UP | 2.00 | chr12 | 9668637 | 9699555 |
| SGCG | 3.62E-02 | DOWN | -1.26 | chr13 | 23180921 | 23325165 |
| RNU6-62P | 1.74E-02 | DOWN | -1.04 | chr13 | 95019235 | 95019336 |
| COL4A2 | 1.27E-06 | DOWN | -2.39 | chr13 | 110305812 | 110513027 |
| DCLK1 | 2.34E-06 | DOWN | -3.37 | chr13 | 35768652 | 36131377 |
| POSTN | 1.97E-03 | DOWN | -7.29 | chr13 | 37562582 | 37609426 |
| COL4A1 | 1.06E-05 | DOWN | -2.08 | chr13 | 110148963 | 110307149 |
| ARHGAP5 | 2.53E-04 | UP | 1.02 | chr14 | 32076967 | 32159728 |
| FRMD6 | 8.52E-05 | DOWN | -3.21 | chr14 | 51489137 | 51730727 |
| DACT1 | 3.13E-03 | UP | 3.02 | chr14 | 58633967 | 58648321 |
| GPR65 | 9.65E-04 | DOWN | -3.19 | chr14 | 88005124 | 88014811 |
| CCNB1IP1 | 3.54E-03 | UP | 1.87 | chr14 | 20311368 | 20333298 |
| RPPH1 | 8.47E-04 | DOWN | -1.15 | chr14 | 20343048 | 20343685 |
| NID2 | 3.20E-05 | DOWN | -3.56 | chr14 | 52004802 | 52069827 |
| RAB15 | 6.22E-04 | DOWN | -1.49 | chr14 | 64945814 | 64972414 |
| FBLN5 | 1.85E-04 | DOWN | -2.04 | chr14 | 91869411 | 91947823 |
| CGNL1 | 2.99E-04 | UP | 1.32 | chr15 | 57376505 | 57550727 |
| LOXL1 | 1.20E-02 | DOWN | -1.18 | chr15 | 73926448 | 73952137 |
| CRABP1 | 9.97E-04 | DOWN | -1.85 | chr15 | 78340324 | 78348231 |
| PLCB2 | 5.66E-04 | DOWN | -3.06 | chr15 | 40287897 | 40307973 |
| FBN1 | 2.56E-05 | DOWN | -2.32 | chr15 | 48408306 | 48645849 |
| IGDCC4 | 1.29E-02 | DOWN | -1.00 | chr15 | 65381464 | 65423072 |
| CAPN3 | 4.13E-03 | UP | 1.05 | chr15 | 42348103 | 42412317 |
| CDH13 | 3.88E-03 | DOWN | -1.26 | chr16 | 82626794 | 83796610 |
| RRAD | 3.31E-03 | DOWN | -1.19 | chr16 | 66921679 | 66925644 |
| TPPP3 | 3.36E-03 | DOWN | -3.41 | chr16 | 67389809 | 67393535 |
| SGCA | 1.13E-02 | UP | 1.29 | chr17 | 50164214 | 50175932 |
| EVI2B | 2.91E-03 | DOWN | -2.90 | chr17 | 31303770 | 31314112 |
| GJC1 | 1.58E-04 | DOWN | -2.12 | chr17 | 44798448 | 44830811 |
| ABCA9 | 1.16E-03 | UP | 1.69 | chr17 | 68974488 | 69060995 |
| ARHGAP28 | 2.16E-03 | DOWN | -2.46 | chr18 | 6729822 | 6915716 |
| DTNA | 6.15E-04 | DOWN | -1.77 | chr18 | 34493290 | 34891844 |
| SLC14A1 | 3.33E-02 | UP | 1.29 | chr18 | 45724127 | 45752520 |
| DSEL | 2.48E-02 | DOWN | -1.07 | chr18 | 67506582 | 67516980 |
| TSHZ2 | 1.06E-04 | DOWN | -2.76 | chr20 | 52972407 | 53495330 |
| SULF2 | 7.48E-04 | UP | 1.50 | chr20 | 47656348 | 47786616 |
| COL6A1 | 2.16E-03 | DOWN | -3.24 | chr21 | 45981737 | 46005050 |
| COL6A2 | 5.55E-03 | DOWN | -2.16 | chr21 | 46098097 | 46132849 |
| ITGB2 | 2.96E-02 | DOWN | -1.57 | chr21 | 44885953 | 44931989 |
| NCF4 | 2.20E-03 | DOWN | -1.97 | chr22 | 36860988 | 36878017 |
| LGALS1 | 1.83E-04 | DOWN | -2.63 | chr22 | 37675606 | 37679806 |
